# Molecular mechanisms of action and prediction of response to oxaliplatin in colorectal cancer cells

**DOI:** 10.1038/sj.bjc.6602215

**Published:** 2004-11-16

**Authors:** D Arango, A J Wilson, Q Shi, G A Corner, M J Arañes, C Nicholas, M Lesser, J M Mariadason, L H Augenlicht

**Affiliations:** 1 Oncology Department, Albert Einstein Cancer Center, Montefiore Medical Center, 111 East 210th St, Bronx, NY 10467, USA; 2 Department of Medical Genetics, Biomedicum Helsinki, PO Box 63 (Haartmaninkatu 8), FIN-00014 University of Helsinki, Finland; 3 School of Public Health, New York Medical College, Valhalla, New York, USA; 4 Biostatistics Unit, North Shore-Long Island Jewish Research Institute, Manhasset, NY, USA

**Keywords:** oxaliplatin, apoptosis, Bax, p53, colorectal cancer, microarray

## Abstract

The platinum compound oxaliplatin has been shown to be an effective chemotherapeutic agent for the treatment of colorectal cancer. In this study, we investigate the molecular mechanisms of action of oxaliplatin to identify means of predicting response to this agent. Exposure of colon cancer cells to oxaliplatin resulted in G2/M arrest and apoptosis. Immunofluorescent staining demonstrated that the apoptotic cascade initiated by oxaliplatin is characterised by translocation of Bax to the mitochondria and cytochrome *c* release into the cytosol. Oxaliplatin treatment resulted in caspase 3 activation and oxaliplatin-induced apoptosis was abrogated by inhibition of caspase activity with z-VAD-fmk, but was independent of Fas/FasL association. Targeted inactivation of Bax or p53 in HCT116 cells resulted in significantly increased resistance to oxaliplatin. However, the mutational status of p53 was unable to predict response to oxaliplatin in a panel of 30 different colorectal cancer cell lines. In contrast, the expression profile of these 30 cell lines, assessed using a 9216-sequence cDNA microarray, successfully predicted the apoptotic response to oxaliplatin. A leave-one-out cross-validation approach was used to demonstrate a significant correlation between experimentally observed and expression profile predicted apoptosis in response to clinically achievable doses of oxaliplatin (*R*=0.53; *P*=0.002). In addition, these microarray experiments identified several genes involved in control of apoptosis and DNA damage repair that were significantly correlated with response to oxaliplatin.

Oxaliplatin is a third generation diaminocyclohexane (DACH) platinum compound that forms mainly intrastrand links between two adjacent guanine residues or a guanine and an adenine, disrupting DNA replication and transcription (Fink *et al*, 1997). Although the related platinum compounds, cisplatin and carboplatin, are generally ineffective in the treatment of colorectal cancer, oxaliplatin has been shown to be effective in the treatment of this disease and it is commonly used to treat patients unresponsive to 5-fluorouracil (5FU) based therapy. However, the details underlying the cytotoxic effects of oxaliplatin remain poorly understood.

Exposure of tumour cells to several chemotherapeutic agents, including oxaliplatin, has been shown to induce programmed cell death or apoptosis (Searle *et al*, 1975; Lowe and Lin, 2000; Gourdier *et al*, 2002; Johnstone *et al*, 2002). In mammalian cells, the signalling cascades leading to apoptosis can be divided into two broad groups. The intrinsic pathway is characterised by the central role of the mitochondria in the initiation of the caspase cascade executing the apoptotic program (Desagher and Martinou, 2000). Pro- and antiapoptotic Bcl-2 family members play a pivotal role in the intrinsic apoptotic cascade (Narita *et al*, 1998; Gross *et al*, 1999). Upon exposure to some apoptotic stimuli, proapoptotic Bcl-2 family members such as Bax, which normally reside in the cytosol, are relocated to the outer mitochondrial membrane where they lead to the release of apoptotic factors from the mitochondria and ultimately to caspase activation and an apoptotic cell death (Li *et al*, 1997; Zou *et al*, 1997). The tumour suppressor gene p53 has been shown to directly regulate the expression levels of Bax (Miyashita and Reed, 1995), and both p53 and Bax have been shown to be important determinants of the cellular response to chemotherapeutic agents (Bunz *et al*, 1999; Zhang *et al*, 2000). In the *extrinsic* pathway, however, caspase activation is initiated by death receptors on the cell surface (Ashkenazi and Dixit, 1998). Chemotherapeutic agents are known to induce apoptosis by either of these two mechanisms (Lowe and Lin, 2000; Johnstone *et al*, 2002).

While significant progress has been made in the identification of markers predicting response to 5FU and CPT-11, two additional chemotherapeutic agents commonly used for colorectal cancer treatment (Augenlicht *et al*, 1997; Bunz *et al*, 1999; Salonga *et al*, 2000; Arango and Augenlicht, 2001; Arango *et al*, 2001; 2003), there is great need for markers that allow discrimination of tumours that vary in their sensitivity to oxaliplatin. The tumour suppressor p53 has a pivotal role in determining the cellular sensitivity to a number of chemotherapeutic agents, including 5FU and CPT-11 (Bunz *et al*, 1999; Arango *et al*, 2001; 2003; Magrini *et al*, 2002). In this study we investigated the potential of this genetic marker to predict response to oxaliplatin *in vitro*. Previous studies have shown that simultaneous analysis of multiple independent markers capable of predicting response to chemotherapy significantly improves the accuracy of the predictions (Salonga *et al*, 2000; Arango *et al*, 2001). cDNA microarray analysis allows the assessment of the level of expression of thousands of genes simultaneously, and its potential to predict response of tumour cells to chemotherapeutic agents has been recently demonstrated (Scherf *et al*, 2000; Kihara *et al*, 2001; Zembutsu *et al*, 2002; Mariadason *et al*, 2003). Therefore, we hypothesised that the expression profile of untreated tumour cells could be used to predict sensitivity to oxaliplatin, and used a leave-one-out cross-validation approach to formally demonstrate the accuracy of this approach in a panel of 30 colorectal cancer cell lines.

## MATERIALS AND METHODS

### Cell culture

HCT116 colon carcinoma cells and isogenic derivatives with a targeted inactivation of p53 or Bax (Bunz *et al*, 1998; Zhang *et al*, 2000) were a gift of Dr Vogelstein (Johns Hopkins University School of Medicine). Cells were maintained in minimum essential medium (MEM) supplemented with 10% fetal bovine serum (FBS), 1 × antibiotic/antimycotic (100 U ml^−1^ streptomycin sulphate, 100 U ml^−1^ penicillin G sodium and 0.25 *μ*g ml^−1^ amphotericin B), 100 *μ*M nonessential amino acids and 10 mM HEPES buffer solution (all from Invitrogen Corporation, Carlsbad, CA, USA).

### Cell cycle and apoptosis

For analysis of cell cycle, 2 × 10^5^ HCT116 cells were seeded on six-well plates in triplicate and allowed to attach for 24 h. For time-response studies cells were treated with 5 or 10 *μ*M oxaliplatin (supplied by Sanofi-Synthelabo, New York, NY, USA) for 12, 24, 48 and 72 h. For concentration–response experiments, cells were treated for 72 h with 5, 10, 15, 20, 25 and 50*μ*M oxaliplatin. Both attached and floating cells were harvested, washed twice with 2 ml of PBS and resuspended in PBS containing 50 *μ*g ml^−1^ propidium iodine (PI), 0.1% sodium citrate and 0.1% Triton X-100. Cells were stained overnight at 4°C and a minimum of 10 000 cells analysed for DNA content using a Becton Dickinson FACScan (Becton Dickinson Immunocytometry Systems, San Jose, CA, USA). The proportion of cells in G0/G1, S phase and G2/M were quantified using ModFit 2.0 (Verity Software House, Topsahm, ME, USA). Apoptosis induced by oxaliplatin treatment was assessed by the nuclear morphology of floating cells. HCT116 cells (1.6 × 10^6^) were seeded on T75 culture flasks and allowed to attach to the substrate for 24 h before exposure to 25 *μ*M oxaliplatin for 48 h. Floating cells were collected, pelleted and re-suspended in 1 *μ*M DAPI (Sigma, St Louis, MO, USA) in PBS. Micrographs of DAPI stained nuclei were captured with a SPOT RT Diagnostic Instruments CCD camera (Diagnostic Instruments, Sterling Heights, MI, USA) attached to a BX60 Olympus fluorescence microscope (Olympus, Melville, NY, USA). Quantification of apoptosis was carried out by PI staining and flow cytometric analysis of the proportion of cells with a subdiploid content of DNA using WinList 2.0 (Verity Software House, Topsahm, ME, USA). In some experiments, cell cultures were preincubated with 2 *μ*g ml^−1^ anti-Fas receptor antibody ZB4 (Upstate Biotechnology, Lake Placid, NY, USA) or 1 *μ*g ml^−1^ anti-Fas ligand antibody NOK-1 (BD Biosciences Pharmingen, San Diego, CA, USA) for 1 h before oxaliplatin cotreatment. Some HCT116 cultures were coincubated with the pan-Caspase inhibitor z-VAD-fmk (Calbiochem, La Jolla, CA, USA) at the final concentrations indicated in the text.

### Caspase 3 activation

HCT116 cells (2 × 10^5^) were seeded on six-well plates in triplicate, allowed to attach for 24 h, and then treated with 25 *μ*M oxaliplatin. After 72 h of treatment, floating cells were collected and pooled with the attached cells harvested by trypsinisation. Cells were washed with PBS, resuspended in CytoFix/CytoPerm (Pharmingen, San Diego, CA, USA), incubated for 30 min on ice and then washed with Perm/Wash buffer (Pharmingen, San Diego, CA, USA) and resuspended in 100 *μ*l of Perm/Wash buffer. In total, 20 *μ*l of phycoerythrin (PE)-conjugated anti-active caspase-3 (Pharmingen, San Diego, CA, USA) were added and cells were incubated at 4°C in the dark for 30 min, washed with Perm/Wash buffer and resuspended in 400 *μ*l of Perm/Wash buffer. Samples were analysed using a Becton Dickinson FACScan, measuring logarithmic PE fluorescence in the FL-2 channel in a minimum of 10 000 cells.

### Western blotting

HCT116 cells seeded in 75 cm^2^ flasks were treated with 10 *μ*M oxaliplatin for 0, 6, 12, 16 or 24 h and then rinsed with PBS twice, harvested and resuspended in 300 *μ*l of RIPA lysis buffer (1% NP-40, 1% sodium deoxycholate, 0.1% SDS, 0.15 M NaCl, 0.01 M sodium phosphate pH 7.2, 2 mM EDTA, 50 mM sodium fluoride, 0.2 mM sodium vanadate and 100 U ml^−1^ aprotinin). The cell suspension was vortexed and kept on ice for 30 min before cell debris was pelleted and the supernatant transferred to a new tube. SDS–polyacrylamide gel electrophoresis sample loading buffer (6 ×) was added to 20 *μ*g aliquots and loaded on 15% tris-HCl precast gels (BioRad, Hercules, CA, USA). Fractionated proteins were transferred to a PVDF membrane (Amersham, Piscataway, NJ, USA) and blocked with 10% nonfat milk for 1 h. Membranes were then probed at room temperature with the appropriate primary antibody in 5% nonfat milk for 1 h with the following antibodies: anti-p53 (Santa Cruz, DO-1, 1/7000), anti-p21^Cip1/WAF1^ (Santa Cruz, H-164, 1/200) and anti-*β*-actin (Sigma, clone AC74, 1/1000). Membranes were washed three times with washing buffer (PBS with 0.1% Tween 20) and then probed with the appropriate peroxidase-conjugated secondary antibody for 1 h (all from Roche Diagnostics/Boehringer Mannheim Corporation, Indianapolis, IN, USA). The secondary antibody was washed three times with washing buffer and the signal was developed using ECL Plus Western Blotting Detection Method (Amersham, Piscataway, NJ, USA). Detection was carried out using a Storm PhosphorImager and quantified using ImageQuant software (Molecular Dynamics, Sunnyvale, CA, USA). Protein levels were standardised using the signal from the *β*-actin probe.

### Immunofluorescence analysis

Cells were cultured overnight on preferred glass coverslips (Fisher, Pittsburgh, PA, USA), and then treated with 10, 20 or 50 *μ*M oxaliplatin. The cells were fixed for 15 min in 4% paraformaldehyde (Electron Microscopy Services, Ft Washington, PA, USA), permeabilised with 0.5% Triton X-100/PBS for 5 min and then incubated for 1 h in a 1% BSA/PBS blocking solution. To detect BAX, cells were incubated for 3 h with a rabbit polyclonal IgG that recognised the N-terminal region (Upstate Biotechnology, Lake Placid, NY, USA; 1 : 100 dilution), followed by exposure to a goat Cy3-conjugated anti-rabbit secondary antibody (Amersham, Piscataway, NJ, USA). To detect mitochondria, a mouse monoclonal Hsp60 antibody was used (Santa Cruz Biotechnology, Santa Cruz, CA, USA; 1 : 200), the binding of which was detected by a goat anti-mouse FITC-conjugated secondary antibody (Roche Diagnostics/Boehringer Mannheim Corporation, Indianapolis, IN, USA). Cytochrome *c* was detected utilising a mouse monoclonal anti-cytochrome *c* IgG (Pharmingen, San Diego, CA, USA; 1 : 200) followed by exposure to a goat anti-mouse Cy5-conjugated secondary antibody (Amersham Biosciences, Piscataway, NJ, USA). All secondary antibodies were used at a dilution of 1 : 200 for 1 h. All washes were performed with PBS. To visualise nuclei, cells were stained with DAPI (Sigma, St Louis, MO, USA; 4′,6-diamidino-2-phenylindole). Fluorescent images were captured with a SPOT RT Diagnostic Instruments CCD camera (Diagnostic Instruments, Sterling Heights, MI, USA) attached to a BX60 Olympus fluorescence microscope (Olympus, Melville, NY, USA). The proportion of apoptotic cells was quantified by scoring the number of cells simultaneously exhibiting Bax relocalisation and cytochrome *c* release in 200 cells.

### Growth/cytotoxicity assay

The concentration of oxaliplatin resulting in 50% inhibition of control growth (GI_50_) in response to oxaliplatin was calculated using the sulphorhodamine B method according to the protocol used by the NCI *in vitro* Anticancer Drug Discovery Screen Program (Skehan *et al*, 1990). Ten thousand cells per well were seeded in 96-well plates and 24 h later exposed to 0, 0.0125, 0.025, 0.125, 0.25, 1.25, 2.5, 6.25, 12.5, 25, 50 and 125 *μ*M oxaliplatin for 72 h. The GI_50_ concentrations were calculated as described (Skehan *et al*, 1990; Arango *et al*, 2003; Mariadason *et al*, 2003) using Prism 3.0 software (GraphPad, San Diego, CA, USA).

### Clonogenic assay

One million cells were seeded in T25 flasks and 24 h later exposed to 0, 2.5, 3, 3.5 or 4 *μ*M oxaliplatin for 9 h. Cells were then trypsinised and 500 cells reseeded in six-well plates in triplicate. Colonies were allowed to form for 2 weeks and then plates were washed and air-dried. Colonies were stained with 0.1% crystal violet, washed three times with distilled water, and air-dried. Plates were scanned using a Perfection 1250 scanner (Epson America Inc., Long Beach, CA, USA) and the number of colonies quantified using Total Lab 1.1 software (Nonlinear Dynamics, Durham, NC, USA).

### Assessment of p53 status

The mutational status of the 30 colorectal cancer cell lines in the panel used has been previously reported (Mariadason *et al*, 2003). Sequencing of the hotspots for p53 mutations (exons 5–8) in T84 cells found no mutations (Mariadason *et al*, 2003). Moreover, Western blot analysis demonstrated that T84 cells have low basal p53 levels, which is characteristic of colorectal cancer cells with a wild-type p53 gene. However, subsequent analyses showed that, unlike other wild-type p53 cell lines in the panel, T84 cells failed to upregulate the p53 target gene p21^Waf1/Cip1^ in response to cytotoxic insult (5 and 50 *μ*M 5-FU for 24 h – not shown). We, therefore, sequenced the remaining of the p53 coding sequence in this cell line and found a missense mutation in exon 4 (codon 60, CCA → CTA; Pro → Leu). Moreover, this mutation has previously been identified in human malignancies, further suggesting that this mutation disrupts p53 function (http://p53.curie.fr). The p53 primers used in the PCR reactions were exon 2: CGACTGTCCAGCTTTGTGC and CCCGTGACTCAGAGAGGACT; exon 2: GGGTTGGAAGTGTCTCATGC and TCCCACAGGTCTCTGCTAGG; exon 3: CAGTCAGATCCTAGCGTCGAG and AGCCCAACCCTTGTCCTTAC; exon 4: CCTCTGACTGCTCTTTTCACC and AGAAATGCAGGGGGATACG; exon 9: GCAGTTATGCCTCAGATTCACT and AACTTTCCACTTGATAAGAGGTC; exon 10: GTACTGTGAATATACTTACTTCT and CCTATGGCTTTCCAACCTAGGAA; exon 11: TTAGGCCCTTCAAAGCATTGGT0 and CACCTATTGCAAGCAAGGGTTCA.

### Microarray analysis

The expression profile of the same panel of 30 colorectal cancer cell lines was assessed in using 9216-sequence cDNA microarrays from the Albert Einstein Cancer Center Facility as previously described (Mariadason *et al*, 2003). For each cell line, hybridisations were carried out in duplicate starting from RNA isolated from independent cultures. For each set of replicates, the mean expression value for each sequence was determined and entered into a final database for further analyses. The expression data for the 3725 sequences with a significant level of expression (defined as signal>background plus two standard deviations in the Cy5 and/or Cy3 channel) in at least one replicate for all 30 cell lines was used in subsequent analyses. All the databases are available on our website (www.augenlichtlab.com).

### ‘Leave-One-Out’ cross-validation analysis

All leave-one-out analyses (Efron and Tibshirani, 1993) were performed using genes that showed a significant level of expression above background in each of the 30 cell lines (3725 of the 9216 genes on the arrays). First, from the 30 cell lines, cell line 1 was removed from consideration, leaving 29 cell lines for analysis. For these 29 cell lines, the Pearson correlation between the level of expression of each of the 3725 genes and the percentage of apoptosis induced by 10 *μ*M oxaliplatin was computed, and the *N* genes highest absolute value correlations (i.e., corresponding to *N* genes) were selected. *N* was varied from the 10 to 200 best-correlated genes. As a control, *N* randomly selected genes were also analysed. To reduce the number of genes to a smaller set of variables, principal components analysis (PCA) was performed. PCA enables a large number of variables to be reduced to linear combinations of variables that can be used to predict an outcome. From the PCA, the principal components (PCs) having the 10 largest eigenvalues were selected. In general, these 10 PCs accounted for approximately 60% of the variance in the selected genes. Next a multiple regression model was developed using the 10 PCs to predict apoptosis, based on the 29 cell lines in the analysis. Once the regression equation was derived, the 10 PCs corresponding to the ‘left out’ cell line were computed and substituted into the derived regression equation to yield a prediction of apoptosis in the left out cell line. Thus, the final results for this first leave-one-out procedure were the predicted value of apoptosis for the left out cell line (*y*_1_^*^) and the observed value (*y*_1_).

After this first leave-one-out procedure was completed, the left out cell line was replaced in the data set, and cell line 2 was removed, once again leaving 29 cell lines in the data set with 1 cell line left out. The entire procedure was repeated for all 30 cell lines so that the final result was a set of predicted apoptosis values for each cell line that had been left out and the corresponding observed value. Each of these 30 leave-one-out procedures yielded 30 pairs of predicted and observed apoptosis values: *y*_1_^*^, *y*_1_, *y*_2_^*^, *y*_2_, …, *y*_30_^*^, *y*_30_.

To determine how well a given regression model predicted observed apoptosis in the left out cell line, the natural log of observed apoptosis (ln(*y*_i_)) was plotted as a function of the natural log of the predicted value (ln(*y*_i_^*^)), and a simple linear regression was constructed. The purpose of this regression analysis was to determine whether the predicted and observed values obeyed the equation *y*_i_=*y*_i_^*^ (i.e., whether the points fall on the line of equality). If the prediction rule is true, then the observed and predicted values would be equal or nearly equal. The measure of linear fit was *r*, and the hypothesis of falling on the line of equality was tested by comparing the slope to unity and *y* intercept to zero.

## RESULTS

### Oxaliplatin induces a G2/M cell cycle arrest and apoptosis

**Figure 1:**
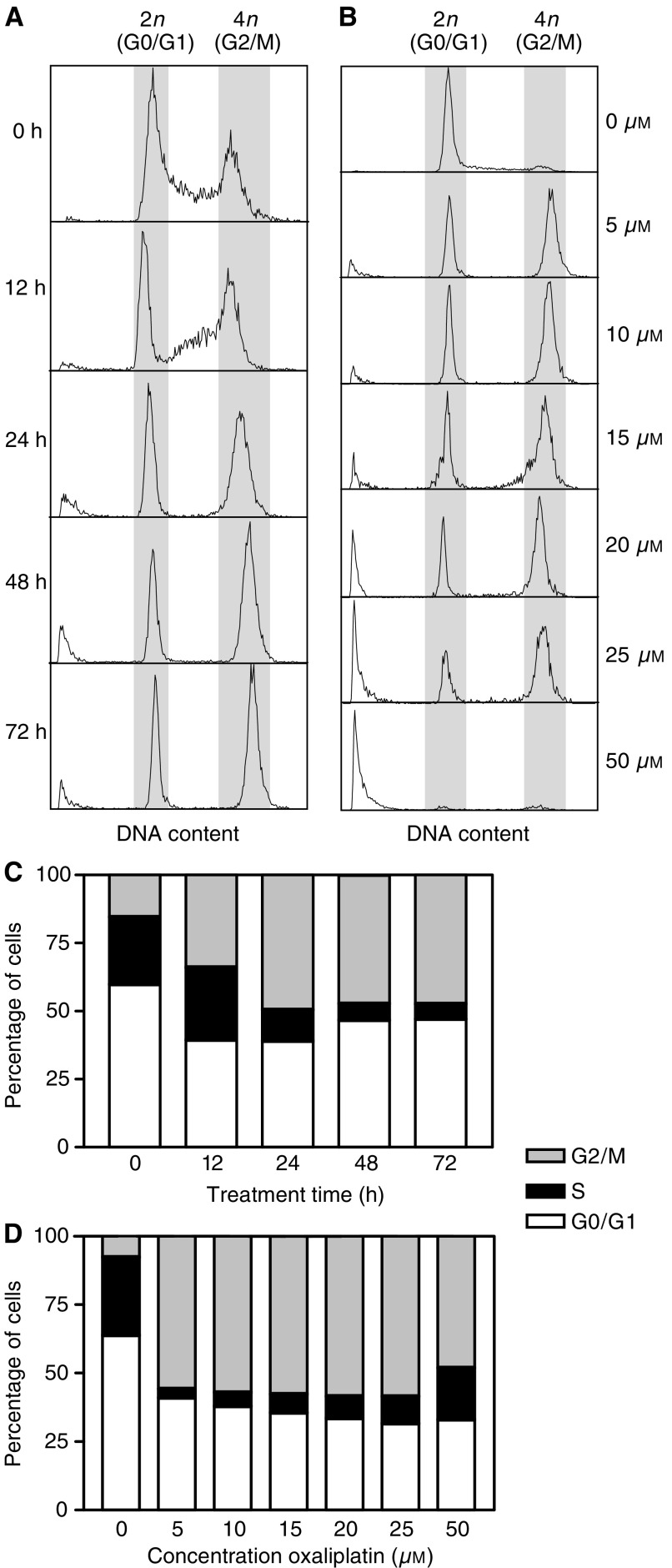
Effects of oxaliplatin on cell cycle. The cell cycle distribution of HCT116 cells was determined after, (**A**) exposure to 5 *μ*M oxaliplatin for different times, and (**B**) treatment for 72 h with different concentrations of oxaliplatin. Representative experiments are shown. In panels (**C** and **D**) the number of cells in G0/G1, S phase and G2/M were quantified by PI staining and flow cytometric analysis. Mean of three experiments is shown.

**Figure 2:**
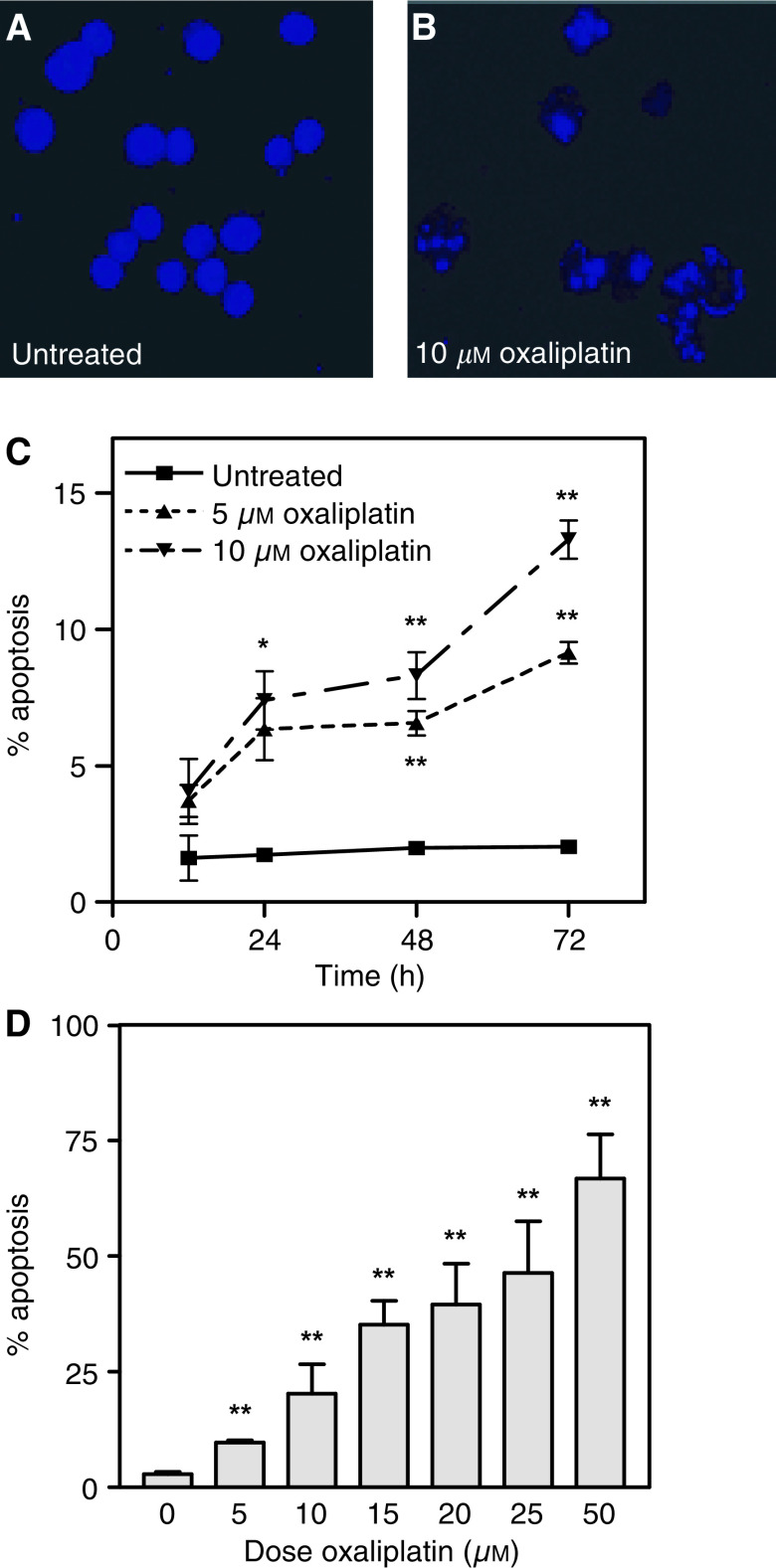
Induction of apoptosis by oxaliplatin. (**A**) Nuclear morphology of DAPI stained untreated HCT116 cells and (**B**) floating cells from cultures treated with 10 *μ*M oxaliplatin for 48 h. The number of apoptotic cells was quantified by PI staining and flow cytometric analysis in cultures treated with 5 or 10 *μ*M oxaliplatin for different times (**C**) and with different concentrations after 72 h of treatment (**D**). Mean of three experiments±s.e. of the mean is shown in (**C** and **D**). ^*^P<0.05 and ^**^P<0.005 (Student's *t*-test).

Exposure of an asynchronous culture of HCT116 colon carcinoma cells to clinically achievable concentrations (Ehrsson *et al*, 2002; Tassone *et al*, 2002) of the platinum compound oxaliplatin resulted in a significant time- and concentration-dependent accumulation of cells in the G2/M phases of the cell cycle and a reduction of cells in S phase (Figure 1A–D

). These effects of oxaliplatin on cell cycle progression of proliferating HCT116 cells were accompanied by a significant proportion of cells showing signs of an apoptotic death, which was confirmed by the characteristic morphological changes observed in DAPI-stained nuclei of oxaliplatin-treated cells (Figure 2A and B

). Quantification of the proportion of apoptotic cells by PI staining and flow cytometric analysis demonstrated that exposure of HCT116 cells to clinically achievable concentrations of oxaliplatin resulted in a significant time-dependent induction of apoptosis (Figures 1A and 2C) and that the proportion of apoptotic cells was concentration-dependent (Figures 1B and 2D).

### The apoptotic cascade initiated by oxaliplatin is characterised by translocation of Bax to the mitochondria, cytochrome *c* release and caspase 3 activation

**Figure 3:**
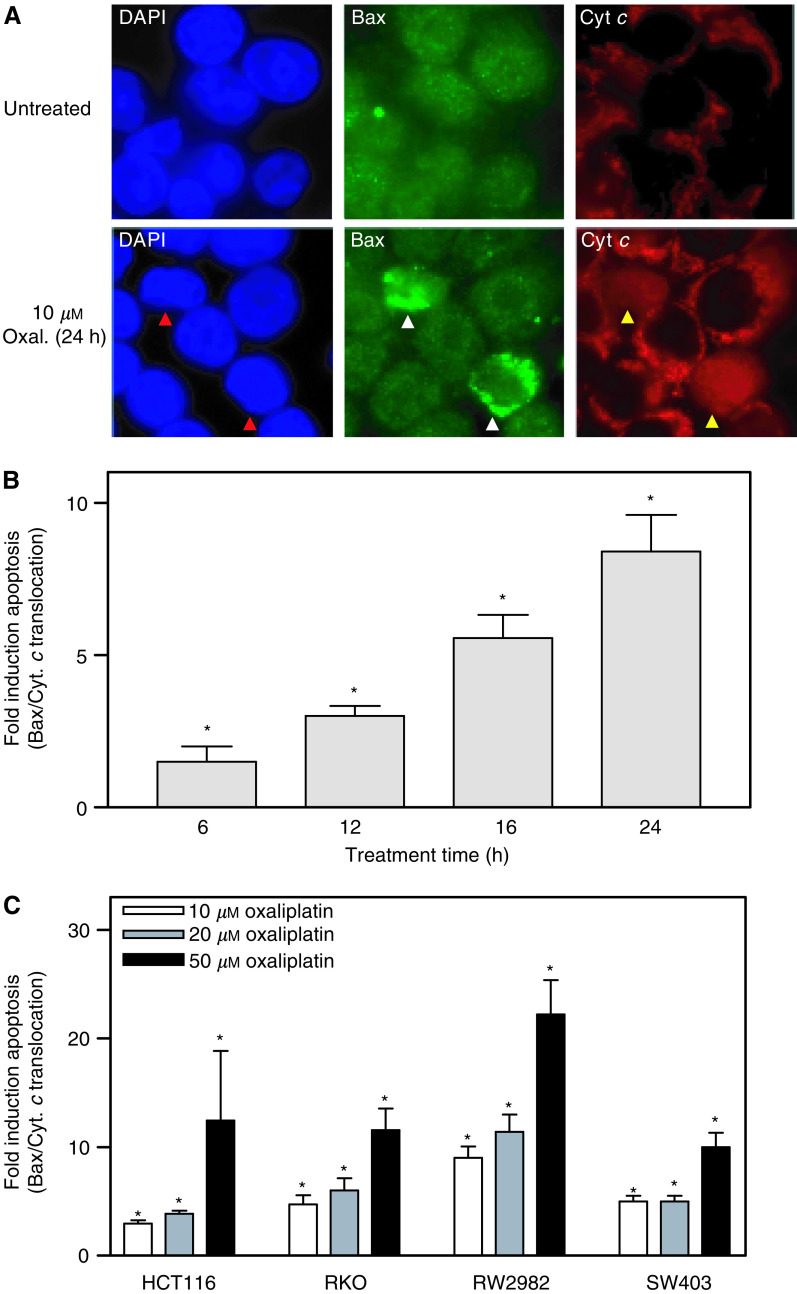
Oxaliplatin induced apoptosis is characterised by Bax relocalisation and cytochrome *c* release. (**A**) Immunofluorescent staining with a Bax antibody demonstrating diffuse cytoplasmic staining in the majority of the cells in untreated cultures is shown. Exposure to 10 *μ*M oxaliplatin for 24 h resulted in a significant proportion of cells exhibiting punctate Bax staining (white arrowheads) consistent with its mitochondrial localisation (see Results). Cytochrome *c* was confined to the mitochondria in the majority of untreated cells. Oxaliplatin treatment resulted in a significant increase in the number of cells displaying a diffuse cytosolic staining of cytochrome *c* (yellow arrowheads). (**B**) Fold induction in the number of cells exhibiting simultaneous Bax relocalisation and cytochrome *c* release in cultures treated with 50 *μ*M oxaliplatin for various times (mean of at least two experiments±s.e.). (**C**) Treatment of HCT116, RKO, RW2982 and SW403 cells with different concentrations of oxaliplatin for 24 h resulted in a significant concentration-dependent increase in the number of cells showing Bax and cytochrome *c* re-localisation. ^*^P<0.01 (Student's *t*-test).

To investigate the molecular cascade of events following exposure to oxaliplatin, we utilised HCT116 cells, which have a wild-type p53 gene and express a functional Bax protein. Immunofluorescent staining of Bax in untreated HCT116 cells demonstrated a diffuse cytoplasmic localisation of Bax in most cells (Figure 3A

). In agreement with a low incidence (<1%) of spontaneous apoptosis in untreated cultures (see Figure 2C), a small proportion of cells showed punctate Bax staining that was demonstrated to localise to the mitochondria by co-staining with Hsp60, a heat shock protein with mitochondrial localisation (Supplementary material Figure 1). However, exposure of HCT116 cells to 10 *μ*M oxaliplatin resulted in a significant (*P*<0.001) increase in the number of cells that exhibited a mitochondrial pattern of Bax staining (white arrowheads in Figure 3A). Translocation of Bax to the mitochondria has been implicated in the formation of the permeability transition pore and cytochrome *c* release from the mitochondria into the cytoplasm (Narita *et al*, 1998; Gross *et al*, 1999). Nonapoptotic untreated HCT116 cells exhibited a mitochondrial localisation of cytochrome *c* (Figure 3A). However, oxaliplatin treatment resulted in an increase in the number of cells showing a diffuse cytoplasmic cytochrome *c* immunostaining (yellow arrowheads in Figure 3A). The observed relocalisation of Bax and cytochrome *c* following treatment of HCT116 cells with oxaliplatin was concurrent (Figure 3A) and time- and concentration-dependent (Figure 3B and C). Extension of these analyses to other colorectal cancer cell lines showed that exposure of RKO, RW2982 and SW403 cells to 10, 20 or 50 *μ*M oxaliplatin resulted in a significant (*P*<0.01) increase in the number of cells showing Bax/cytochrome *c* re-localisation (Figure 3C).

**Figure 4:**
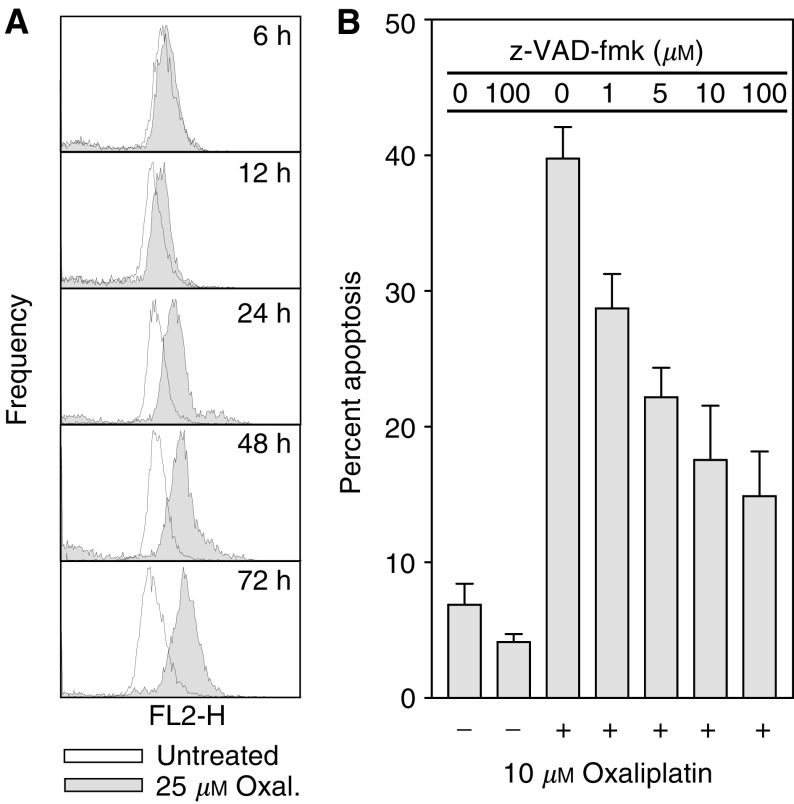
Caspase 3 activation in oxaliplatin treated cells. (**A**) FACS analysis of HCT116 cells stained with a PE-conjugated antibody specific to active Caspase 3 demonstrates that oxaliplatin treatment induces a time-dependent increase of active Caspase 3. (**B**) Oxaliplatin-induced apoptosis was abrogated in a dose-dependent manner by the caspase inhibitor z-VAD-fmk. Mean of three experiments±s.e. of the mean.

Cytoplasmic cytochrome *c* has been shown to bind to other components of the apoptosome, resulting in caspase activation (Liu *et al*, 1996; Zou *et al*, 1997). A time-dependent Caspase 3 activation was demonstrated in oxaliplatin-treated cells by immunostaining with a fluorescently labelled antibody that binds specifically to active Caspase 3, and quantified using flow cytometry (Figure 4A

). In agreement with this observation, treatment of HCT116 cells with oxaliplatin in the presence of different concentrations of z-VAD-fmk, a pan-caspase inhibitor, reduced oxaliplatin-induced apoptosis in a concentration-dependent manner (Figure 4B).

### Targeted inactivation of Bax decreased the apoptotic response to oxaliplatin

**Figure 5:**
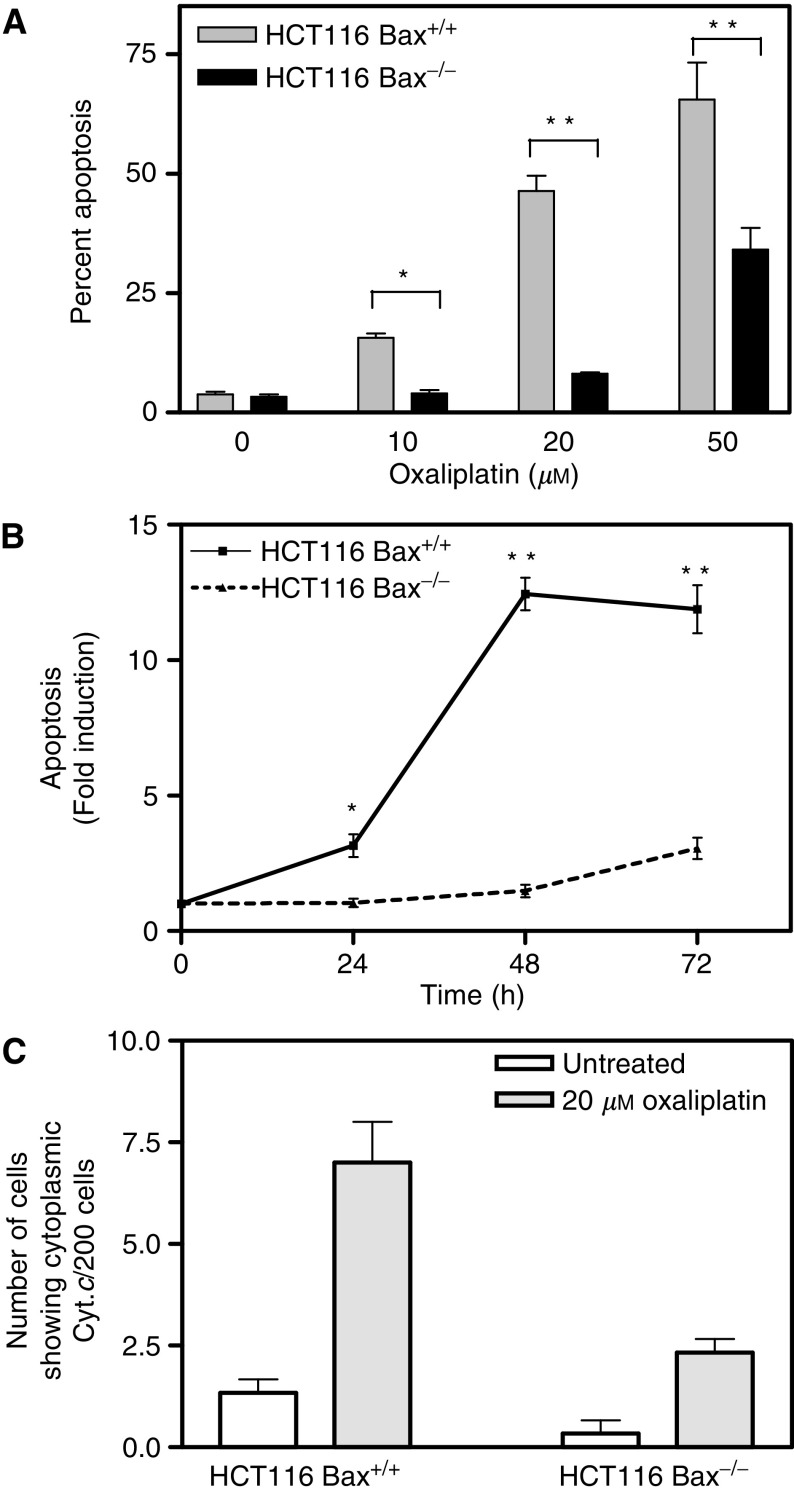
Role of Bax in sensitivity of colon cancer cells to oxaliplatin. (**A**) Percentage of apoptotic cells following exposure of HCT116 Bax^+/+^ and Bax^−/−^ to the indicated concentrations of oxaliplatin for 72 h is shown. (**B**) Induction of apoptosis in isogenic Bax proficient and deficient HCT116 cells after exposure to 20 *μ*M oxaliplatin at various times is shown. (**C**) Number of cells with a cytoplasmic cytochrome *c* staining pattern (per 200 cells) following exposure to 20 *μ*M oxaliplatin for 24 h. In all cases, the mean of three experiments±s.e. of the mean is shown. ^*^P<0.05; ^**^P<0.01 (Student's *t*-test).

The cellular translocation of Bax to the mitochondria observed in HCT116 cells following exposure to oxaliplatin suggested a functional role for this Bcl2 family member in the apoptotic cascade initiated by oxaliplatin. To investigate the role of Bax in oxaliplatin-induced apoptosis, we utilised an isogenic HCT116 derivative line that differs only in the absence of a functional Bax gene. Propidium iodide (PI) staining and flow cytometric analysis demonstrated a significant (*P*<0.03) time- and dose-dependent reduction in the number of apoptotic cells in HCT116 Bax^−/−^ cells compared to isogenic Bax^+/+^ cells following oxaliplatin exposure (Figure 5A and B

). In agreement with this observation, HCT116 Bax^−/−^ cells treated with 20 *μ*M oxaliplatin for 24 h showed a significant (*P*=0.03) reduction compared to isogenic Bax^+/+^ cells in the number of cells with the cytosolic staining pattern of cytochrome *c* characteristic of apoptotic cells (Figure 5C), further demonstrating an important functional role of Bax in oxaliplatin-induced apoptosis.

### Oxaliplatin-induced apoptosis is not dependent upon Fas/FasL association in HCT116 cells

**Figure 6:**
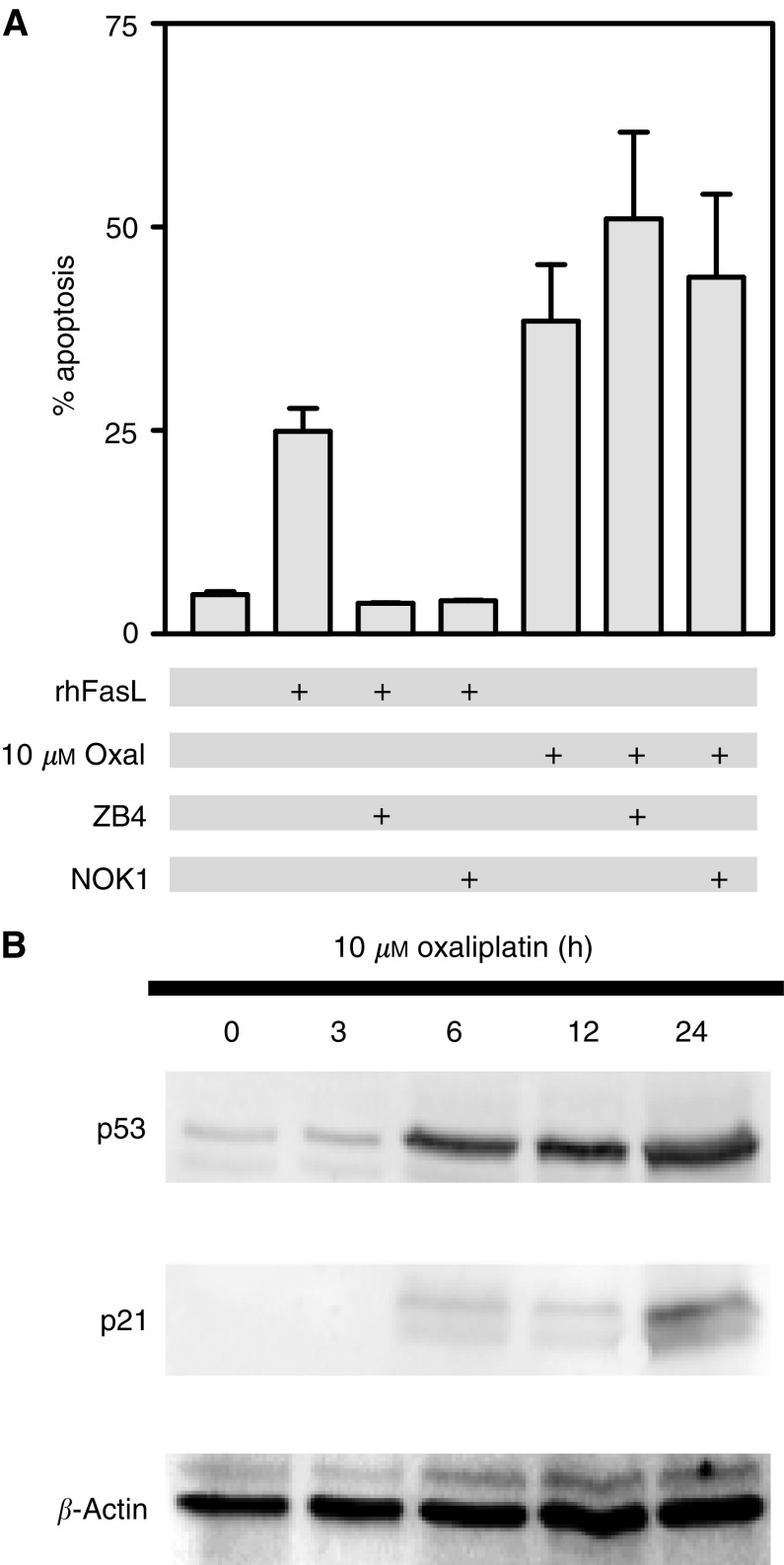
(**A**) Role of Fas/FasL association in oxaliplatin-induced apoptosis. Exposure of HCT116 to either recombinant human Fas ligand (rhFasL) or oxaliplatin resulted in significant induction of apoptosis. Preincubation with antibodies that prevent Fas/FasL association (ZB4 or NOK-1) for 1 h prevented apoptosis induced by rhFasL but had no effects on oxaliplatin-induced apoptosis. Mean of three experiments±s.e. (**B**) Effects of oxaliplatin treatment in p53 levels. Western blot analysis demonstrated that exposure of HCT116 cells to 10 *μ*M oxaliplatin results in increased levels of p53 and the cdk inhibitor p21^waf1/cip1^, a p53 target gene. *β*-Actin levels on a parallel blot are shown as a loading control.

Bax relocalisation to the mitochondria, accumulation of cytochrome *c* in the cytoplasm and Caspase 3 activation are all events consistent with an intrinsic pathway of activation of apoptosis by oxaliplatin. To investigate the contribution of the extrinsic pathway in oxaliplatin-induced apoptosis, we used antibodies that specifically recognise either the Fas receptor (ZB4) or the Fas ligand (NOK-1) and disrupt Fas/FasL association and the subsequent induction of apoptosis by this pathway. Preincubation of HCT116 cells with either ZB4 or NOK-1 antibodies was effective in preventing apoptosis induced by recombinant human soluble Fas ligand (rhFasL; Figure 6A

). However, pretreatment with ZB4 or NOK-1 antibodies did not affect apoptosis induced by oxaliplatin (Figure 6A), suggesting that this extrinsic pathway of induction of apoptosis was not activated by oxaliplatin in HCT116 cells.

### Role of p53 in the response to oxaliplatin

**Figure 7:**
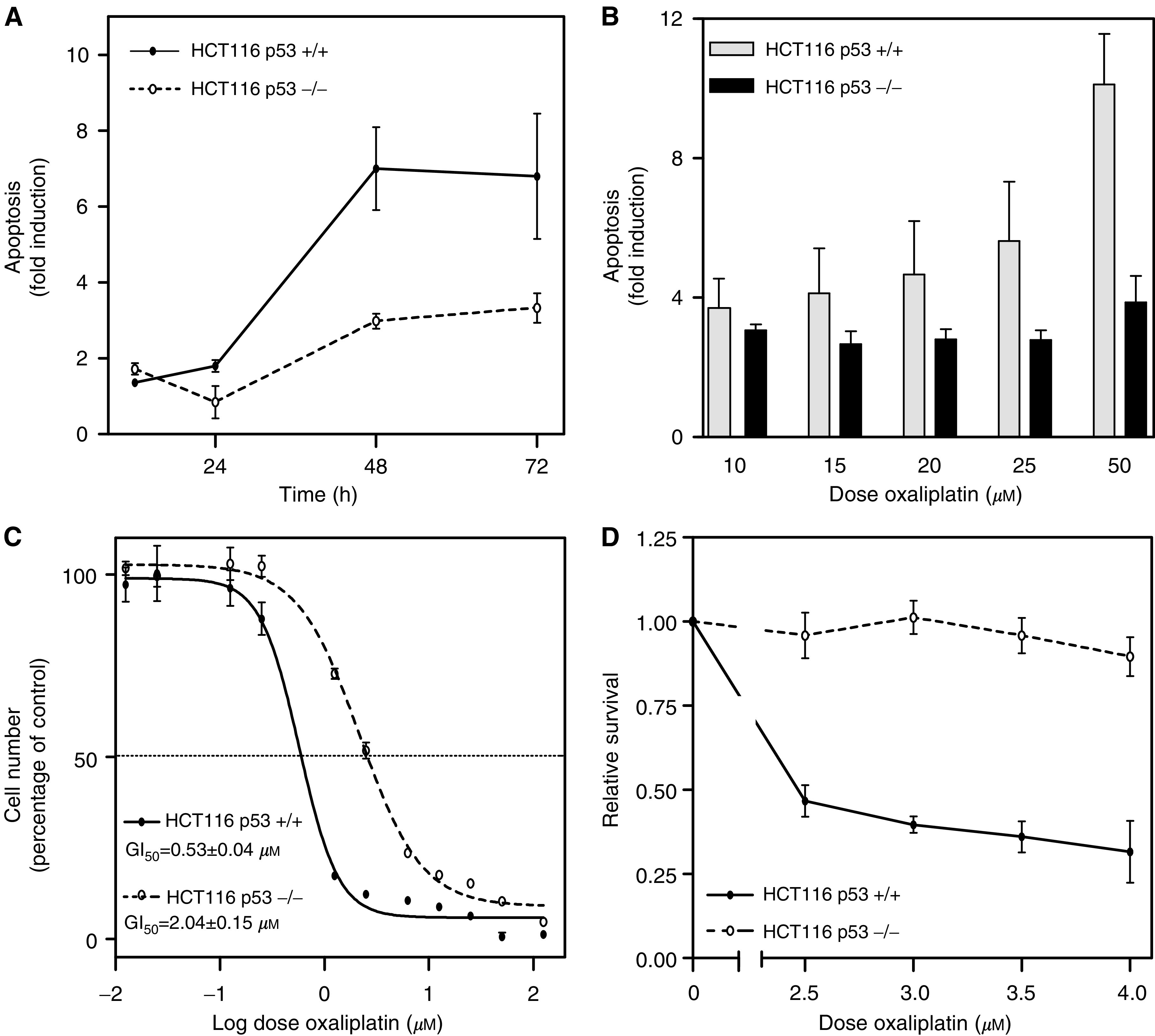
Role of p53 in sensitivity of colon cancer cells to oxaliplatin. (**A**) Fold induction of apoptosis in HCT116 p53^+/+^ and HCT116 p53^−/−^ cultures after exposure to 25 *μ*M oxaliplatin for different times, or (**B**) to different concentrations oxaliplatin for 72 h are shown. (**C**) Comparison of oxaliplatin-induced growth inhibition between HCT116 p53^+/+^ and p53^−/−^ cells. (**D**) Clonogenic potential of HCT116 p53^+/+^ and p53^−/−^ cells treated with 2.5–4 *μ*M oxaliplatin for 9 h. Values shown are the mean of at least three different experiments±s.e. of the mean.

More than 50% of colonic tumours have a mutant p53 gene (Baker *et al*, 1990), and a functional p53 protein has been shown to be important for the cellular response to a variety of proapoptotic stimuli, including chemotherapeutic agents commonly used in the treatment of colorectal cancer, such as 5FU and CPT-11 (Bunz *et al*, 1999; Arango *et al*, 2001; 2003; Magrini *et al*, 2002). Here, we demonstrate that exposure of wild-type p53 HCT116 cells to oxaliplatin results in increased levels of p53 (Figure 6B), and consistent with this observation, the p53 target gene p21^waf1/cip1^ was also upregulated (Figure 6B). The product of the p53 gene is a transcription factor that can either promote apoptosis, through different mechanisms such as Bax upregulation, or induce cell cycle arrest and DNA damage repair by means of the transcriptional upregulation of genes such as p21^waf1/cip1^ and Gadd45. Therefore, we directly tested the role of p53 in the sensitivity of colon cancer cells to oxaliplatin using parental wild-type p53 HCT116 cells and isogenic HCT116 p53^−/−^ cells. Relative to parental cells, p53-deficient HCT116 cells showed a time- and concentration-dependent protection from the apoptotic effects of oxaliplatin (Figure 7A and B

). The reduced apoptotic effects of oxaliplatin in HCT116 p53^−/−^ could be detected after 16 h of treatment as a 10-fold reduction in the number of cells exhibiting Bax translocation and cytochrome *c* release compared to parental HCT116 p53^+/+^ (not shown). Moreover, the concentration of oxaliplatin necessary to cause a 50% growth inhibition (GI_50_) after 72 h of exposure was four-fold higher in HCT116 p53^−/−^ cells compared to parental HCT116 cells (2.04±0.15 and 0.53±0.04 *μ*M respectively; *P*<0.0001; Figure 7C). To investigate the long-term implications of the reduced apoptosis and growth inhibition in response to oxaliplatin in HCT116 p53^−/−^ cells, we assessed the clonogenic potential of parental HCT116 p53^+/+^ and isogenic HCT116 p53^−/−^ cells 2 weeks after exposure to oxaliplatin for 9 h. This assay demonstrated that exposure of parental HCT116 p53^+/+^ cells to concentrations of oxaliplatin ranging from 2.5 to 4 *μ*M, resulted in up to 70% reduction in the number of cells with long-term clonogenic potential. In contrast, these concentrations of oxaliplatin had no effect on clonogenicity of HCT116 p53^−/−^ cells (Figure 7D), further demonstrating that inactivation of p53 in colon cancer cells results in a significant protection from oxaliplatin cytotoxicity.

**Figure 8:**
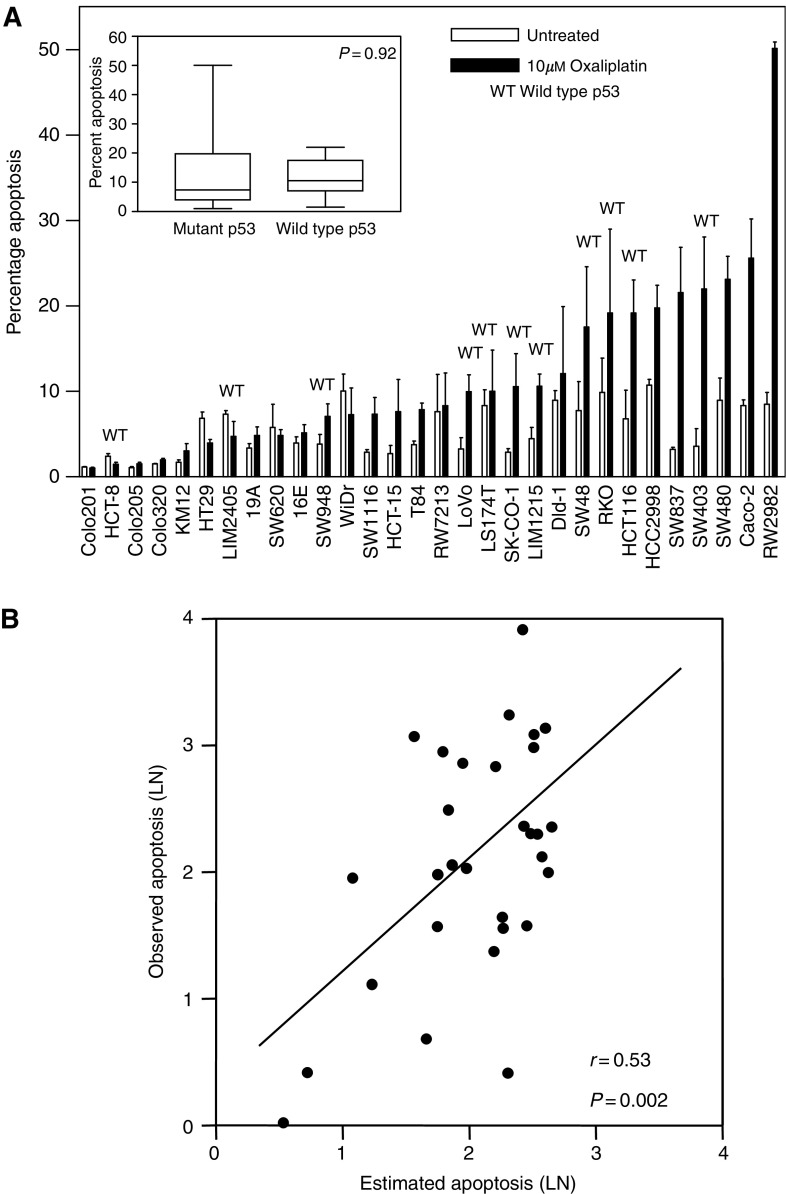
Microarray-based prediction of response to oxaliplatin. (**A**) Percentage of apoptotic cells following exposure of a panel of 30 colorectal cancer cell lines to 10 *μ*M oxaliplatin for 72 h. Cell lines with a wild-type p53 gene are indicated (WT). The inset shows the mean percentage apoptosis in p53 wild type and mutant cell lines. Mean of three independent experiments in triplicate±s.e. of the mean is shown. (**B**) Correlation between experimentally observed and expression profile predicted percentage apoptosis following treatment with 10 *μ*M oxaliplatin for 72 h. The predicted apoptosis value for all 30 cell lines was calculated using the expression profile of the 80 genes best correlated with drug response and a multiple regression model through a leave-one-out cross-validation approach (see Material and Methods).

To further investigate the role of p53 in the response of colorectal cancer cells to oxaliplatin, we assessed the sensitivity to this agent in a panel of 30 different colorectal cancer cell lines of known p53 mutational status (Mariadason *et al*, 2003). Of these 30 cell lines, 11 had a wild-type p53 gene, and the remaining 19 exhibited inactivating mutations in this tumour suppressor. Considerable variability was observed among different colorectal cancer cell lines in the number of apoptotic cells following 72 h treatment with 10 *μ*M oxaliplatin (Figure 8A

). Despite the clear role of p53 in the apoptotic response to oxaliplatin demonstrated using the HCT116 isogenic system that differs only in the presence or absence of a functional p53 protein, the mutational status of this tumour suppressor gene could not predict the apoptotic response to 10 *μ*M oxaliplatin (Figure 8A), suggesting that additional factors modulate sensitivity to this agent.

### Response to oxaliplatin can be predicted using the expression profile of untreated colorectal cancer cells

Due to the great complexity of the molecular mechanisms determining response to oxaliplatin, analysis of the p53 mutational status is likely to be of limited predictive value in the clinical setting. As an alternative approach, we used cDNA microarray analysis to assess the levels of expression of 9216 sequences in the same panel of 30 colorectal cancer cell lines, and used the expression profile of untreated cells to make predictions concerning response to oxaliplatin.

In order to validate the accuracy of the predictions within the panel of 30 cell lines we used a ‘leave-one-out’ cross-validation approach. Here, one of the 30 samples is held out, and the *N* genes whose expression best correlates with the apoptotic response are selected using the remaining 29 cell lines. The apoptotic response in the 30th line is then estimated using the expression of those *N* genes and a predictor based on a multiple regression model (see Materials and Methods for details). This process is repeated 30 times holding out a different cell line in each iteration. In this way, a predicted value for the apoptotic response to oxaliplatin is obtained for each of the 30 cell lines, which can then be compared to the experimentally observed response to this agent. To optimise the predictive rule, we tested the effect of varying the number of *N* input genes from the 10-best through 200-best correlated with oxaliplatin-induced apoptosis (see Materials and Methods). This analysis demonstrated that selection of the 80 genes best correlated with oxaliplatin-induced apoptosis produced the most accurate prediction, with a highly significant correlation (*r*=0.53, *P*=0.002) between the observed and estimated response to oxaliplatin (Figure 8B). Importantly, the correlation between observed and estimated response to oxaliplatin by a group of 80 randomly selected genes was not significant (*P*=0.19). This formally demonstrates that cDNA microarray based expression profiling can be used to predict response to oxaliplatin in colorectal cancer cells.

The ‘leave-one-out’ cross-validation approach adopted produced a slightly different list of 80 genes in each iteration, as a different cell line is held out in each round of analysis. A total of 254 genes were used in at least one of the 30 cycles, and 28 of them were present in all of the 30 gene lists used (Table 1

**Table 1:** List of 254 genes used at least once in the 30 leave-one-out cross-validation loops

**Accession**	**Name**	**Correlation^a^**	**Count in LOOCV^b^**	**Biological process (GO)^c^**
AA455003	General transcription factor IIH, polypeptide 1, 62 kDa	−0.77	30	DNA repair
R98008	Progestin and adipoQ receptor family member III	−0.70	30	Biological process unknown
AA417012	Zinc-finger protein 336	−0.67	30	Regulation of transcription, DNA-dependent
AA412691	Nuclear transcription factor Y, alpha	−0.67	30	Regulation of transcription, DNA-dependent
AA030029	Protein kinase C, alpha	−0.65	30	Regulation of cell cycle
R06567	Clone 23907 mRNA sequence	−0.64	30	Biological process unknown
AA521025	G protein pathway suppressor 1	−0.62	30	JNK cascade
AA405769	Phosphoenolpyruvate carboxykinase 1 (soluble)	−0.62	30	Gluconeogenesis
AA608576	Microtubule-associated protein, RP/EB family, member 2	−0.62	30	Cell proliferation
AA083385	BTB (POZ) domain containing 1	−0.62	30	Biological process unknown
W32660	RAP2A, member of RAS oncogene family	−0.61	30	Signal transduction
R46662	RAP2A, member of RAS oncogene family	−0.61	30	Signal transduction
H14343	Cell division cycle 25B	−0.60	30	M phase of mitotic cell cycle
AA480071	Transcription factor 7 (T-cell specific, HMG-box)	−0.60	29	Wnt receptor signaling pathway
AA456014	Hypothetical protein FLJ20580	−0.60	30	Biological process unknown
H09906	2′,3′-cyclic nucleotide 3′ phosphodiesterase	−0.60	30	Synaptic transmission
N51260	KIAA0240	−0.60	29	Biological process unknown
H56069	Glutamate–cysteine ligase, catalytic subunit	−0.59	30	Cysteine metabolism
AA489104	Thyroid hormone receptor interactor 3	−0.59	29	Regulation of transcription, DNA-dependent
T77959	Tripeptidyl peptidase II	−0.59	29	Proteolysis and peptidolysis
AA479196	General transcription factor IIF, polypeptide 2, 30 kDa	−0.59	30	Transcription initiation from Pol II promoter
AA430656	Docking protein 4	−0.58	29	Biological process unknown
H30688	Spectrin, beta, nonerythrocytic 2	−0.58	29	Vesicle-mediated transport
R10570	Hypothetical protein KIAA1434	−0.58	28	Biological process unknown
R54358	Ligase IV, DNA, ATP-dependent	−0.57	27	DNA repair
H08548	ATP citrate lyase	−0.57	27	Coenzyme A biosynthesis
AA418811	Fibrillin 1 (Marfan syndrome)	−0.57	26	Skeletal development
AA410591	Met proto-oncogene (hepatocyte growth factor receptor)	−0.57	28	Cell proliferation
W86653	FK506-binding protein 5	−0.56	27	Protein folding
H11501	Hypothetical protein BC017169	−0.56	27	Transport
AA521490	Limkain b1	−0.56	27	Biological process unknown
AA447691	Pitrilysin metalloproteinase 1	−0.56	27	Activation of MAP/ERK kinase kinase
R42187	ESTs	−0.56	27	Biological process unknown
H89996	CCCTC-binding factor (zinc-finger protein)	−0.56	27	Negative regulation of cell cycle
AA148536	Nucleoporin 98 kDa	−0.56	25	Intracellular protein transport
R01323	Microfibrillar-associated protein 1	−0.56	25	Biological process unknown
AA425401	Serine/threonine kinase 24 (STE20 homolog, yeast)	−0.55	25	Signal transduction
T49159	Serine (or cysteine) proteinase inhibitor, clade B (ovalbumin), member 2	−0.55	24	Antiapoptosis
AA001918	ESTs	−0.55	24	Biological process unknown
N70759	KIAA0996 protein	−0.55	24	Biological process unknown
AA232979	Zinc-finger RNA binding protein	−0.55	19	Biological process unknown
AA521232	ras-related C3 botulinum toxin substrate 2	−0.55	20	Small GTPase mediated signal transduction
AA426311	Mesenchyme homeo box 1	−0.55	21	Development
AA074666	ESTs	−0.54	15	Biological process unknown
AA479888	Rabaptin, RAB GTPase binding effector protein 1	−0.54	14	Endocytosis
AA701929	Bystin-like	−0.54	15	Cell adhesion
AA479771	KIAA0076	−0.54	19	Biological process unknown
R76314	ras homolog gene family, member G (rho G)	−0.54	15	Positive regulation of cell proliferation
AA004484	F-box only protein 9	−0.54	17	Protein modification
AA458840	Cell death-regulatory protein GRIM19	−0.54	9	Apoptosis
AA036974	Amine oxidase, copper containing 3	−0.54	9	Amine metabolism
R28280	Solute carrier family 7, member 1	−0.54	10	Amino acid metabolism
AA045192	Retinoblastoma 1 (including osteosarcoma)	−0.53	8	Negative regulation of cell cycle
H99215	Discs, large homolog 3 (neuroendocrine-dlg, Drosophila)	−0.53	10	Negative regulation of cell proliferation
T71757	Heme oxygenase (decycling) 1	−0.53	6	Heme oxidation
AA453175	Bridging integrator 1	−0.53	7	Negative regulation of cell cycle
R12275	Hypothetical protein FLJ21827	−0.53	6	Biological process unknown
AA478524	M-phase phosphoprotein 6	−0.53	7	M phase of mitotic cell cycle
AA434342	Proline arginine-rich end leucine-rich repeat protein	−0.53	6	Skeletal development
AA459266	Postmeiotic segregation increased 2-like 6	−0.53	5	Mismatch repair
H49592	Guanine nucleotide binding protein (G protein), alpha activating activity polypeptide	−0.53	7	G-protein coupled receptor protein signaling pathway
R99749	B-cell CLL/lymphoma 6 (zinc-finger protein 51)	−0.53	7	Cell growth and/or maintenance
AA621031	KIAA0056 protein	−0.53	7	Biological process unknown
AA232647	Zinc-finger protein 161	−0.52	5	Regulation of transcription from Pol II promoter
AA485214	Nucleobindin 2	−0.52	5	Biological process unknown
AA458973	Polymerase (DNA-directed), delta interacting protein 3	−0.52	3	Biological process unknown
AA608730	HBS1-like (*S. cerevisiae*)	−0.52	3	Protein biosynthesis
H98688	ESTs	−0.52	4	Biological process unknown
AA425908	ADP-ribosylation factor interacting protein 2 (arfaptin 2)	−0.52	3	Small GTPase mediated signal transduction
AA424937	Protein kinase C, alpha	−0.52	4	Regulation of cell cycle
AA017042	HIV-1 Tat interactive protein, 60 kDa	−0.52	3	Regulation of transcription, DNA-dependent
AA284338	Chromobox homolog 5 (HP1 alpha homolog, Drosophila)	−0.51	2	Chromatin assembly
AA040170	Chemokine (C-C motif) ligand 7	−0.51	3	Antimicrobial humoral response
AA425089	Clock homolog (mouse)	−0.51	4	Circadian rhythm
N58136	Similar to KIAA0393 protein	−0.51	2	Biological process unknown
R24543	Neuroepithelial cell transforming gene 1	−0.51	2	Regulation of cell growth
R15708	Insulin-like 4 (placenta)	−0.51	2	Cell proliferation
W47073	Solute carrier family 20 (phosphate transporter), member 1	−0.51	3	Phosphate transport
AA703187	Solute carrier family 33 (acetyl-CoA transporter), member 1	−0.51	4	Transport
W79444	UBX domain containing 2	−0.51	2	Biological process unknown
R35292	Growth arrest-specific 2 like 1	−0.51	3	Cell cycle arrest
AA406027	CD5 antigen (p56-62)	−0.51	2	Cell proliferation
W06875	Hypothetical protein MGC14816	−0.51	2	Biological process unknown
R14855	F-box only protein 34	−0.50	2	Biological process unknown
R01451	Hypothetical protein KIAA1259	−0.50	1	Biological process unknown
AA449154	Skeletal muscle and kidney enriched inositol phosphatase	−0.50	2	Actin cytoskeleton organization and biogenesis
H48420	Prothymosin alpha	−0.50	1	Biological process unknown
AA455043	Holocarboxylase synthetase	−0.50	2	Protein modification
AA232647	Zinc-finger protein 161	−0.50	2	Regulation of transcription from Pol II promoter
R11490	Translocated promoter region (to activated MET oncogene)	−0.50	2	Transport
T58146	HLA complex P5	−0.50	2	Defense response
H54366	KIAA0034 gene, complete cds	−0.50	2	Biological process unknown
AA406269	Nuclear factor I/X (CCAAT-binding transcription factor)	−0.50	2	DNA replication
R02346	Small nuclear ribonucleoprotein 70 kDa polypeptide (RNP antigen)	−0.50	1	RNA splicing
H64850	Clone 23612 mRNA sequence	−0.50	1	Biological process unknown
N74574	Hypothetical protein MGC9084	−0.49	3	Biological process unknown
N26645	Neurofibromin 1 (neurofibromatosis, von Recklinghausen disease, Watson disease)	−0.49	2	RAS protein signal transduction
R26434	Protein phosphatase 1, catalytic subunit, beta isoform	−0.49	1	Biological process unknown
AA025850	Myosin VA (heavy polypeptide 12, myoxin)	−0.49	1	Transport
H59208	ESTs	−0.49	1	Biological process unknown
AA490721	Splicing factor, arginine/serine-rich 9	−0.49	2	mRNA splice site selection
R10604	Spinocerebellar ataxia 2 (olivopontocerebellar ataxia 2, autosomal dominant, ataxin 2)	−0.49	1	mRNA splicing
R95732	DNA (cytosine-5-)-methyltransferase 2	−0.49	1	DNA methylation
AA149096	Hemopoietic cell kinase	−0.49	2	Biological process unknown
AA489714	Chromosome 9 open reading frame 60	−0.49	1	Biological process unknown
AA045587	TAF12 RNA polymerase II, TATA box binding protein (TBP)-associated factor, 20 kDa	−0.49	2	Regulation of transcription, DNA-dependent
AA485443	Ring finger protein 41	−0.48	1	Biological process unknown
AA085676	ADP-ribosylation factor guanine nucleotide factor 6	−0.48	1	Biological process unknown
W58032	Frizzled-related protein	−0.48	1	Wnt receptor signaling pathway
N71653	Aspartoacylase (aminoacylase 2, Canavan disease)	−0.48	2	Aspartate catabolism
AA004324	Hypothetical protein BC007706	−0.48	1	Biological process unknown
AA441935	Achaete-scute complex-like 1 (Drosophila)	−0.48	1	Cell differentiation
R22050	Topoisomerase (DNA) II beta (180kD)	−0.48	1	DNA topological change
W31074	Fatty-acid-Coenzyme A ligase, long-chain 3	−0.48	3	Fatty acid metabolism
T72581	Matrix metalloproteinase 9	−0.48	3	Collagen catabolism
AA453105	Histone 1, H2ac	−0.48	1	Nucleosome assembly
AA988746	WD repeat and SOCS box containing protein 2	−0.48	1	Intracellular signaling cascade
AA495766	Chromosome condensation 1-like	−0.48	1	Biological process unknown
AA489699	COP9 constitutive photomorphogenic homolog subunit 8 (Arabidopsis)	−0.48	1	Biological process unknown
T65902	Splicing factor, arginine/serine-rich 1 (splicing factor 2, alternate splicing factor)	−0.48	1	mRNA splice site selection
AA446908	Kinesin family member 3C	−0.47	1	Biological process unknown
AA047812	Origin recognition complex, subunit 2-like (yeast)	−0.47	1	DNA replication
AA452566	Peroxisomal membrane protein 3, 35 kDa (Zellweger syndrome)	−0.47	1	Peroxisome organization and biogenesis
R91948	Pantothenate kinase 3	−0.47	1	Coenzyme A biosynthesis
T52894	Myosin light chain 1 slow a	−0.47	1	Muscle development
AA001749	Microtubule-associated protein, RP/EB family, member 1	−0.47	1	Cell proliferation
AA430035	Reticulon 3	−0.47	1	Biological process unknown
N79179	Pyruvate dehydrogenase complex, lipoyl-containing component X; E3-binding protein	−0.47	1	Biological process unknown
R13911	T54 protein	−0.47	1	Biological process unknown
AA447579	Hypothetical protein FLJ11101	−0.47	1	Biological process unknown
AA454639	F-box only protein 9	−0.47	1	Protein modification
AA476460	Protein tyrosine phosphatase, receptor-type, Z polypeptide 1	−0.46	2	One-carbon compound metabolism
AA147642	cDNA: FLJ21949 fis, clone HEP04922	−0.46	1	Biological process unknown
AA479954	Similar to RNA polymerase B transcription factor 3	−0.46	1	Biological process unknown
N80382	Phosphodiesterase 6D, cGMP-specific, rod, delta	−0.46	1	Visual perception
R88246	Adrenergic, beta, receptor kinase 1	−0.45	1	Signal transduction
AA150895	Hypothetical protein MGC26690	−0.45	1	Biological process unknown
AA680407	Similar to ubiquitin binding protein	−0.45	1	Biological process unknown
AA063398	Chromosome 15 open reading frame 15	−0.45	1	Protein biosynthesis
AA459572	Protein phosphatase 1, regulatory subunit 7	−0.45	1	Biological process unknown
AA401429	Dynein light chain 2	−0.45	2	Microtubule-based process
AA434130	Thioredoxin reductase 2	−0.44	1	Response to oxygen radicals
AA453593	BB1=malignant cell expression-enhanced gene/tumor progression-enhanced gene	−0.44	1	Protein arginylation
T77891	ESTs	−0.44	1	Biological process unknown
N48137	Glycophorin E	−0.44	1	Biological process unknown
N30147	CDNA FLJ34899 fis, clone NT2NE2018594.	−0.43	1	Biological process unknown
AA011185	Hypothetical protein FLJ14431	−0.43	1	Metabolism
N58558	Serine (or cysteine) proteinase inhibitor, clade A (alpha-1 antiproteinase, antitrypsin), member 4	−0.43	1	Acute-phase response
AA055221	Apoptosis, caspase activation inhibitor	−0.43	1	Apoptosis
AA280677	Zinc-finger protein 258	−0.43	1	Development
AA026709	Dedicator of cytokinesis 6	−0.43	1	Biological process unknown
AA487070	Similar to CGI-55 protein (LOC152502), mRNA	−0.43	1	Biological process unknown
AA284282	Hypothetical protein FLJ30277	−0.43	1	Biological process unknown
T57859	Natural killer cell group 7 sequence	−0.42	1	Biological process unknown
AA448001	TBP-like 1	−0.42	1	Regulation of transcription
N92085	ESTs	−0.41	1	Biological process unknown
AA423867	Multimerin	−0.41	1	Blood coagulation
AA443302	ras homolog gene family, member E	−0.38	1	Small GTPase mediated signal transduction
H52361	ESTs	−0.37	1	Biological process unknown
AA482198	Mannose phosphate isomerase	−0.36	1	Carbohydrate metabolism
H66030	Centrosomal protein 1	−0.36	1	Biological process unknown
AA458472	Major histocompatibility complex, class II, DQ beta 1	−0.36	1	Immune response
AA447696	Yippee protein	−0.35	1	Biological process unknown
AA419164	Retinoic acid receptor, beta	−0.34	1	Cell growth and/or maintenance
AA453849	ATP synthase, H+ transporting, mitochondrial F0 complex, subunit b, isoform 1	−0.32	1	ATP synthesis coupled proton transport
T96688	PBX/knotted 1 hoemobox 1	−0.32	1	Biological process unknown
R41732	Mitogen-activated protein kinase 8 interacting protein 1	0.28	1	Vesicle-mediated transport
AA419622	Hypothetical protein FLJ10134	0.31	1	Biological process unknown
AA128017	Clone DNA100312 VSSW1971 (UNQ1971) mRNA	0.33	1	Biological process unknown
R23810	Full-length insert cDNA YI09H09	0.33	1	Biological process unknown
T90871	EST	0.35	1	Biological process unknown
AA005228	ESTs	0.39	1	Biological process unknown
AA004811	ESTs	0.39	1	Biological process unknown
R54797	ESTs, Weakly similar to reverse transcriptase homolog	0.39	1	Biological process unknown
T95930	ESTs	0.40	1	Biological process unknown
AA058713	ESTs	0.41	1	Biological process unknown
T84865	ESTs	0.41	1	Biological process unknown
N46943	GGA binding partner	0.41	1	Biological process unknown
AA677403	Glycoprotein hormones, alpha polypeptide	0.42	1	Cell–cell signaling
AA872379	SMT3 suppressor of mif two 3 homolog 1 (yeast)	0.42	1	Biological process unknown
T96870	ESTs	0.42	1	Biological process unknown
AA610111	Hypothetical protein FLJ21868	0.43	1	Biological process unknown
AA400121	A-kinase anchoring protein 28	0.44	1	Biological process unknown
H72319	ESTs	0.44	1	Biological process unknown
H23124	Olfactomedin related ER localized protein	0.44	1	Biological process unknown
AA447515	MAX dimerization protein 4	0.44	1	Negative regulation of cell proliferation
N62914	EST	0.44	1	Biological process unknown
T53298	Insulin-like growth factor binding protein 7	0.45	1	Negative regulation of cell proliferation
AA421488	CDNA FLJ26472 fis, clone KDN04506	0.46	1	Biological process unknown
H93837	Apolipoprotein B (including Ag(x) antigen)	0.46	1	Lipid transport
N78306	ESTs	0.46	1	Biological process unknown
N32502	ESTs	0.46	1	Biological process unknown
AA488672	Kruppel-like factor 7 (ubiquitous)	0.47	1	Regulation of transcription from Pol II promoter
AA053815	Hypothetical protein FLJ11767	0.47	1	Biological process unknown
R02716	Hypothetical protein FLJ14639	0.47	1	Biological process unknown
AA701545	Ribonuclease, RNase A family, k6	0.47	1	RNA catabolism
R93069	ESTs	0.47	1	Biological process unknown
T54121	Cyclin E1	0.48	2	Regulation of cell cycle
H57309	Snail homolog 2 (Drosophila)	0.48	1	Development
T56948	Pumilio homolog 1 (Drosophila)	0.48	1	Biological process unknown
N93191	Voltage-dependent calcium channel gamma subunit-like protein	0.48	1	Biological process unknown
W33154	Formin binding protein 1	0.49	1	Biological process unknown
T95113	Viperin	0.49	1	Biological process unknown
AA679177	Butyrate-induced transcript 1	0.49	2	Activation of JUNK
W89074	ESTs	0.49	1	Biological process unknown
R97540	Down syndrome critical region gene 3	0.49	2	Intracellular protein transport
W60581	Brain expressed, X-linked 1	0.49	2	Biological process unknown
AA037352	Paired box gene 1	0.50	2	Biological process unknown
T53389	Fc fragment of IgG binding protein	0.50	1	Biological process unknown
N53480	Clone IMAGE:4794941, mRNA	0.50	3	Biological process unknown
AA410604	CDC16 cell division cycle 16 homolog (*S. cerevisiae*)	0.50	1	Cell cycle
N22827	Ferredoxin 1	0.50	1	Steroid metabolism
R95893	EST	0.50	1	Biological process unknown
AA872397	Lectin, galactoside-binding, soluble, 2 (galectin 2)	0.50	2	Heterophilic cell adhesion
H72588	ESTs	0.51	2	Biological process unknown
N92048	ESTs	0.51	1	Biological process unknown
N54793	ESTs	0.51	3	Biological process unknown
AA136699	ESTs	0.51	1	Biological process unknown
AA131466	Leukemia inhibitory factor receptor	0.51	4	Cell surface receptor linked signal transduction
N33331	Peroxisome proliferative activated receptor, delta	0.52	3	Lipid metabolism
AA668527	Mucosal vascular addressin cell adhesion molecule 1	0.52	5	Cell adhesion
AA677706	Lactotransferrin	0.52	3	Iron ion transport
AA459390	Hypothetical protein FLJ22169	0.53	6	Biological process unknown
W02624	Kelch repeat and BTB (POZ) domain containing 2	0.53	6	Biological process unknown
AA984940	Ribonuclease, RNase A family, 2 (liver, eosinophil-derived neurotoxin)	0.53	11	RNA catabolism
AA464180	X-linked protein	0.53	9	Biological process unknown
R89765	Full-length insert cDNA clone YP91F02	0.54	11	Biological process unknown
AA102068	F-box and leucine-rich repeat protein 8	0.54	13	Biological process unknown
AA431429	ESTs, Weakly similar to A-kinase anchor protein DAKAP550 [*D. melanogaster*]	0.54	20	Biological process unknown
AA009671	ESTs	0.54	21	Biological process unknown
AA458965	Natural killer cell transcript 4	0.55	19	Cell adhesion
H51438	ESTs	0.55	24	Biological process unknown
AA447593	Dynein, axonemal, light intermediate polypeptide 1	0.55	26	Cell motility
T98717	ESTs	0.56	26	Biological process unknown
AA485358	Seven transmembrane domain protein	0.56	25	Biological process unknown
AA022935	Formin-like 2	0.56	27	Regulation of transcription, DNA-dependent
H87770	Full-length insert cDNA YI27F12	0.56	25	Biological process unknown
AA024866	Hypothetical protein FLJ32731	0.56	28	Biological process unknown
AA055992	Hypothetical protein MGC17791	0.57	28	Biological process unknown
R92446	Cytokine receptor-like molecule 9	0.57	28	Biological process unknown
AA443971	ESTs	0.57	29	Biological process unknown
N79813	CDNA FLJ45930 fis, clone PLACE7000707	0.59	28	Biological process unknown
W80688	Zinc finger, CW-type with coiled-coil domain 1	0.59	29	Biological process unknown
N72185	ESTs	0.60	30	Biological process unknown
AA136666	CDNA: FLJ22750 fis, clone KAIA0478	0.60	30	Biological process unknown
AA133167	KIAA1644 protein	0.60	30	Biological process unknown
T84703	ESTs	0.61	30	Biological process unknown
AA029997	Collagen, type IV, alpha 5 (Alport syndrome)	0.62	30	Biological process unknown
AA953975	Nuclear factor of kappa light polypeptide gene enhancer in B-cells inhibitor, epsilon	0.62	30	I-kappaB kinase/NF-kappaB cascade
W88801	Trans-prenyltransferase	0.63	30	Isoprenoid biosynthesis
N70632	Alcohol dehydrogenase, iron containing, 1	0.65	30	Metabolism
AA701081	Leucine-rich repeat transmembrane neuronal 2 protein	0.65	30	Biological process unknown
W93482	LOC374841 (LOC374841), mRNA	0.67	30	Biological process unknown
R20886	Stanniocalcin 2	0.68	30	Cell–cell signaling

a Correlation coefficient between expression levels in 30 colorectal cancer cell lines (Ln) and the corresponding proportion of apoptotic cells after 72 h exposure to 10 *μ*M oxaliplatin (Ln). The correlation of all 9216 genes can be found in our website: www.augenlichtlab.com.

b Number of times used in the leave-one-out cross-validation process.

c Biological process from Gene Ontology.

). The expression profile of all 9216 genes in the cDNA microarrays used can be found on our website (www.augenlichtlab.com).

## DISCUSSION

The platinum compound oxaliplatin is frequently used in the treatment of colorectal cancer patients that are resistant to 5FU, and can also be used in combination with 5FU or CPT-11, improving response rates and progression-free survival (Levi *et al*, 1993; Bertheault-Cvitkovic *et al*, 1996; Machover *et al*, 1996; de Gramont *et al*, 1997; 2000; Andre *et al*, 1999; Maindrault-Goebel *et al*, 1999; Giacchetti *et al*, 2000). Oxaliplatin disrupts DNA replication and transcription by forming intrastrand DNA adducts, but the downstream molecular events underlying the cytotoxic effects of this chemotherapeutic agent have not been well characterised.

Here, we show that exposure of HCT116 colon cancer cells to clinically relevant concentrations of oxaliplatin (Ehrsson *et al*, 2002; Tassone *et al*, 2002) greatly reduced the long-term clonogenic potential of these cells (solid line in Figure 7D). This was associated with an arrest of proliferating cells in the G2/M phases of the cell cycle (Figure 1). Consistent with this observation, there was a significant reduction in the growth rate of oxaliplatin-treated cells compared to control untreated cells (solid line in Figure 7C). Importantly, exposure of HCT116 cells to oxaliplatin resulted in a significant induction of apoptosis detectable as early as 24 h after treatment with the lowest concentration assessed (see Figure 2C). Although impairment of the growth of tumour cells is an important component contributing to the overall response to chemotherapy, cell death is the preferred method of elimination of malignant cells, since this is a terminal and irreversible mechanism. Clonal selection of tumour cells frequently results in the acquisition of mechanisms of evading apoptosis. Therefore, understanding of the molecular mechanisms involved in the induction of apoptosis after exposure to chemotherapeutic agents is important for two reasons: first, it can provide information regarding pathways that may be modulated to improve treatment efficacy, and second, it can lead to the identification of markers capable of predicting the probability of response to treatment.

In this study we demonstrate that exposure of four different colorectal cancer cells (HCT116, RKO, RW2982 and SW403) to oxaliplatin resulted in recruitment of Bax to the mitochondria, release of cytochrome *c* to the cytosol and Caspase 3 activation. Targeted inactivation of Bax in HCT116 cells resulted in a significant reduction in the number of cells displaying a cytosolic staining pattern of cytochrome *c* and terminal apoptosis following exposure to oxaliplatin. These results demonstrated an important functional role of Bax in the apoptotic cascade of events initiated by exposure to oxaliplatin and are in agreement with previous reports (Gourdier *et al*, 2002; Hayward *et al*, 2004). Moreover, it has been suggested that frameshift mutations in the G8 track of the Bax gene could contribute to the acquisition of resistance to oxaliplatin in HCT116 cells (Gourdier *et al*., 2002). Collectively, these observations suggest the potential of Bax as a genetic marker capable of predicting the probability of response of colorectal cancer patients to this important chemotherapeutic agent.

Bax relocalisation to the mitochondria, release of cytochrome *c* to the cytosol and Caspase 3 activation, are all events consistent with the induction of an intrinsic apoptotic pathway, characterised by the central role of the mitochondria in the initiation of the caspase cascade. Some chemotherapeutic agents, however, induce an apoptotic response through activation of the extrinsic pathway by promoting Fas receptor/Fas ligand association, which in turn leads to the formation of the death-inducing signaling complex (DISC) and the autocatalytic activation of pro-caspase 8. To investigate the contribution of this pathway to oxaliplatin-induced apoptosis, we utilised antibodies that bind to Fas or FasL, thus preventing the association of these two proteins. Although abrogation of Fas/FasL association completely prevented apoptosis induced by recombinant human Fas ligand, it had no effect on oxaliplatin-induced apoptosis, suggesting the predominance of the intrinsic pathway in the apoptotic program initiated by oxaliplatin exposure in HCT116. However, Marchetti *et al* (2004) have recently demonstrated the involvement of the extrinsic pathway in oxaliplatin-induced apoptosis in HCT15 colon cancer cells, suggesting that the role of this pathway may be tumour dependent.

Considerable progress has been made in the identification of genetic markers allowing prediction of colorectal cancer response to 5-FU and CPT-11, which together with oxaliplatin are commonly used for the treatment of these patients (Augenlicht *et al*, 1997; Bunz *et al*, 1999; Salonga *et al*, 2000; Arango and Augenlicht, 2001; Arango *et al*, 2001; 2003). However, despite recent efforts (Arnould *et al*, 2003; Rakitina *et al*, 2003) it is currently not possible to accurately predict response to oxaliplatin. p53 is mutated in over 50% of colorectal tumours, and the mutational status of this tumour suppressor has been shown to increase or decrease tumour sensitivity to a number of chemotherapeutic agents. Here we show that oxaliplatin-induced apoptosis was associated with upregulation of p53 protein levels, detectable within 6 h of treatment. This suggested a role of p53 in the apoptotic cascade initiated by oxaliplatin. Using an isogenic system we showed that targeted inactivation of p53 resulted in a four-fold increase in the GI_50_, reduced apoptosis and induced a significant increase in clonogenic potential after exposure to oxaliplatin, demonstrating that inactivation of p53 can lead to significantly increased resistance to oxaliplatin.

However, there are some reports showing that p53 inactivation does not lead to increased resistance to oxaliplatin (Seo *et al*, 2002; Petit *et al*, 2003), suggesting that the role of p53 in the cellular response to oxaliplatin may be tumour dependent. To further investigate the role of p53 in sensitivity of colorectal cancer cells to oxaliplatin, we used a panel of 30 different cell lines of known p53 mutational status. We measured the induction of apoptosis following exposure of these 30 cell lines to clinically achievable doses of oxaliplatin, and found that cell lines with a wild type and mutant p53 gene did not significantly differ in their apoptotic response to oxaliplatin (see Figure 8A). Similar disparities have been reported for the role of p53 in 5FU response, depending upon whether isogenic cell lines or panels of colorectal cell lines were studied (Yang *et al*, 1996; Bunz *et al*, 1999; Violette *et al*, 2002). Therefore, despite the clear role of p53 in the response of colon cancer cells to oxaliplatin demonstrated using the HCT116 isogenic system, the multiple genetic and epigenetic differences that exist between tumours are likely to affect numerous pathways, thus modulating sensitivity to oxaliplatin. This could limit the clinical value of p53 and other single markers of response to treatment.

Simultaneous analysis of several independent markers predicting response to drug treatment has been shown to be advantageous over single markers (Salonga *et al*, 2000; Arango *et al*, 2001). Microarray analysis provides the means of assessing the levels of expression of thousands of genes simultaneously, and we and others have recently demonstrated that the expression profile of untreated tumour cells can be used to predict response of tumour cells to different chemotherapeutic agents *in vitro* and *in vivo* (Scherf *et al*, 2000; Kihara *et al*, 2001; Zembutsu *et al*, 2002; Mariadason *et al*, 2003). Here, we used a cDNA microarray-based approach to measure the expression levels of 9216 transcripts in a panel of 30 colorectal cancer cell lines for which the sensitivity to oxaliplatin was also assessed. The profile of expression of the 80 genes best correlated with sensitivity was used to predict the quantitative apoptotic response to oxaliplatin in these 30 cell lines. Using a ‘leave-one-out’ cross-validation approach we demonstrated a highly significant correlation between experimentally observed and microarray-predicted apoptotic response to oxaliplatin (*R*=0.53, *P*=0.002).

Our microarray experiments identified a number of genes and expressed sequence tags (ESTs) that are differentially expressed in cell lines that are sensitive and resistant to oxaliplatin. Among the 254 transcripts that were used in at least one of the 30 ‘leave-one-out’ cross-validation loops, several of them have a known role in apoptosis (Table 1). The serine/threonine kinase Protein Kinase C alpha (PKC*α*) has been associated with cell survival and the suppression of apoptosis (Deacon *et al*, 1997; Orlandi *et al*, 2003). PKC*α* was represented twice in our cDNA microarray and both probes demonstrated that expression levels were lower in cell lines with a higher apoptotic response to oxaliplatin. This is in good agreement with previous reports demonstrating that PKC*α* levels modulate the cellular response to cisplatin and oxaliplatin (Johnson *et al*, 2002; Orlandi *et al*, 2003). The transcription factor NF*κ*B has been shown to be frequently deregulated in colorectal tumours, and it is associated with increased proliferation and resistance to apoptosis induced by chemotherapeutic agents (Garg and Aggarwal, 2002; Orlowski and Baldwin, 2002). Here we found that the NF*κ*B inhibitor epsilon (NFKBIE) is expressed at higher levels in the more sensitive cell lines. This is consistent with a recent report showing that donwregulation of the transcriptional activity of NF*κ*B significantly sensitises colorectal cancer cells to cytotoxic effects of oxaliplatin (Rakitina *et al*, 2003). In addition, the recently identified apoptosis inhibitor AVEN (Chau *et al*, 2000) was found to be expressed at higher levels in cell lines that showed reduced apoptosis in response to oxaliplatin.

The cytotoxic activities of oxaliplatin are believed to be linked to DNA damage, and the levels of expression of the DNA repair endonuclease ERCC1 (excision repair cross-complementing 1) have been shown to be inversely correlated with response to oxaliplatin (Shirota *et al*, 2001; Arnould *et al*, 2003). Although ERCC1 was not represented in the 9K chips used in this study, our microarray analyses identified at least four genes involved in DNA repair mechanisms that were significantly correlated with the ability of oxaliplatin to induce apoptosis in colorectal cancer cells (see Table 1). The histone acetyltransferase HTATIP (HIV-1 Tat interactive protein, 60 kDa), postmeiotic segregation increased 2-like 6 (PMS2L6), general transcription factor IIH, polypeptide 1 (GTF2H1), and the DNA ligase LIG4, showed higher levels of expression in cell lines that are more resistant to oxaliplatin treatment. In fact, at least LIG4 has been shown previously to confer resistance to the related platinum compound cisplatin (Chipitsyna *et al*, 2004).

In summary, this study investigates the molecular mechanisms underlying the cytotoxic effects of oxaliplatin in colorectal cancer cells in an attempt to identify different means of predicting response to this chemotherapeutic agent. We demonstrate that exposure of proliferating colorectal cancer cells to oxaliplatin induces a G2/M arrest and a molecular cascade of events consistent with an intrinsic mechanism of apoptosis. Moreover, the cytotoxic effects of oxaliplatin were shown to be Bax and p53 dependent using and isogenic *in vitro* system. Importantly, we demonstrate that the expression profile of untreated tumour cells can be used to predict response to oxaliplatin, and that this approach outperforms the accuracy of p53 mutational status as a predictive marker. The efficacy of this microarray-based approach to predict response to oxaliplatin remains to be confirmed *in vivo*. Collection of tumour samples from suitable patient populations is currently ongoing at our institution to test the value of these approaches, although completion of the study is dependent upon prolonged follow-up periods to assess response to therapy.

## Supplementary Material

Supplementary Material Figure 1 Cellular localization of Bax and Cytochrome C in control and oxaliplatin treated cells. a) Immunofluorescent staining of Bax in untreated HCT116 cells showed a diffuse cytoplasmic pattern. Exposure to 10 μM oxaliplatin for 24h resulted in re-localization of Bax to the mitochondria in a significant number of cells, as demonstrated by its co-localization with HSP60, a heat shock protein of mitochondrial localization (white arrow head). b) Cytochrome C shows a mitochondrial localization in untreated HCT116. Oxaliplatin treatment resulted in the release of Cytochrome C to the cytosol in a significant number of cells (red arrow head).
