# Supplementary figures and images for: Molecular mechanisms of action and prediction of response to oxaliplatin in colorectal cancer cells
Source: Br J Cancer. 2004 Nov 16;91(11):1931–46. doi: 10.1038/sj.bjc.6602215 (PMC2409767; doi:10.1038/sj.bjc.6602215)

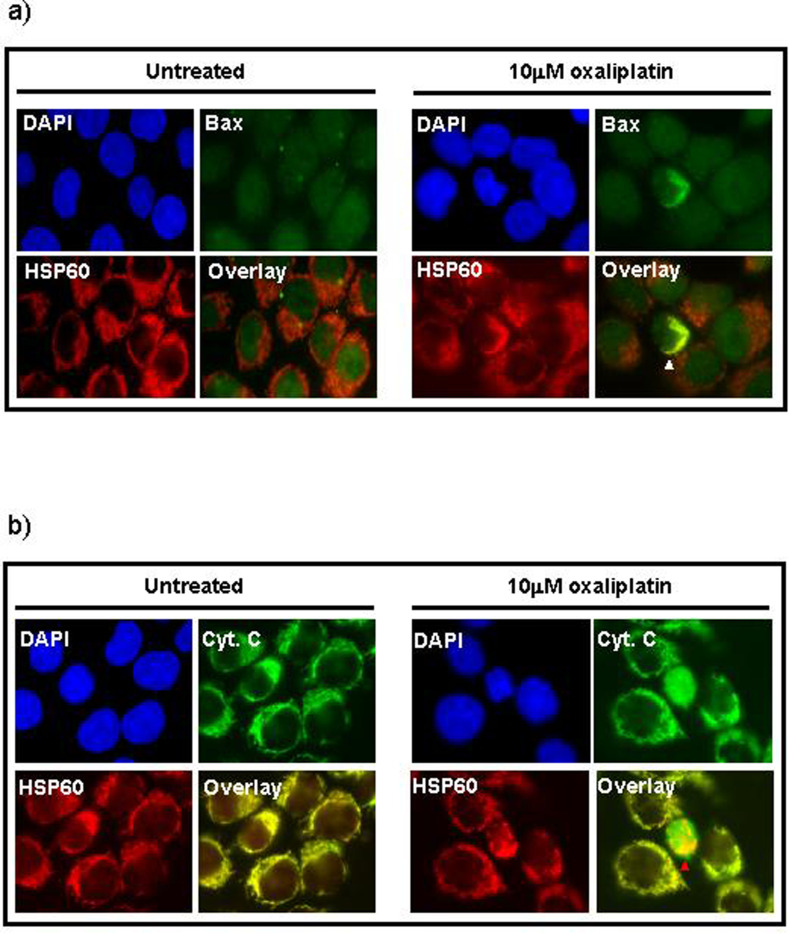

Supplement: Supplementary Material Figure 1 — Cellular localization of Bax and Cytochrome C in control and oxaliplatin treated cells. a) Immunofluorescent staining of Bax in untreated HCT116 cells showed a diffuse cytoplasmic pattern. Exposure to 10 μM oxaliplatin for 24h resulted in re-localization of Bax to the mitochondria in a significant number of cells, as demonstrated by its co-localization with HSP60, a heat shock protein of mitochondrial localization (white arrow head). b) Cytochrome C shows a mitochondrial localization in untreated HCT116. Oxaliplatin treatment resulted in the release of Cytochrome C to the cytosol in a significant number of cells (red arrow head). [file 91-6602215x1.jpg]
